# MiniMovers: An Initial Pilot and Feasibility Study to Investigate the Impact of a Mobile Application on Children’s Motor Skills and Parent Support for Physical Development

**DOI:** 10.3390/children11010099

**Published:** 2024-01-14

**Authors:** Yajie Zhang, Nalda Wainwright, Jacqueline D. Goodway, Amanda John, Anna Stevenson, Kirsty Thomas, Sean Jenkins, Fatma Layas, Kate Piper

**Affiliations:** 1 Assistive Technologies Innovation Centre (ATiC), University of Wales Trinity Saint David, Swansea SA1 8PH, UK; y.zhang@uwtsd.ac.uk (Y.Z.); sean.jenkins@uwtsd.ac.uk (S.J.); f.layas@uwtsd.ac.uk (F.L.); 2 Wales Academy for Health and Physical Literacy, University of Wales Trinity Saint David, Carmarthen SA31 3EP, UK; n.wainwright@uwtsd.ac.uk (N.W.); amanda.john@uwtsd.ac.uk (A.J.); a.stevenson@uwtsd.ac.uk (A.S.); kirsty.edwards@uwtsd.ac.uk (K.T.); 3 Department of Human Sciences, The Ohio State University, Columbus, OH 43210-1172, USA; 4 Sport and Healthy Living, University of Wales Trinity Saint David, Carmarthen SA31 3EP, UK; k.piper1@uwtsd.ac.uk

**Keywords:** physical activity, motor competency, physical literacy, motor skills, egame

## Abstract

The MiniMovers (MM) APP combines motor development theory with creativity expertise and has been designed to provide parents with developmentally appropriate activities to support children’s motor skills. This study investigates how MiniMovers activities enabled parents to support their children’s physical development. Families participated in an 8-week MM programme of activities from the MM APP (Mini, Mighty and Mega levels), with pre- and post-intervention data collected using multiple tools (e.g., motion capture system, force plate, eye-tracking glasses, and videos). Mixed research methods were applied among children (*N* = 8; aged 21–79 months) and their parents, providing quantitative analysis on children’s performance (running, throwing, jumping, kicking, balancing and catching), as well as qualitative analysis on parents’ attitude and behaviour (two-weekly feedback surveys and interviews). Lab-based measures showed significant improvements in run time, underarm throwing distance, and horizontal jump distance. Test of Gross Motor Development-3 showed a significant gain in running, underarm and overarm throwing, horizontal jump and kicking. Further, developmental stages indicated significant improvements in running, kicking and catching. Parents reported increased enjoyment and knowledge, children’s enjoyment, independence and confidence. This pilot study provides support for the research and development of the MM App and suggests more research into the use of APPs to support home activities among families with young children.

## 1. Introduction

Physical activity in childhood is associated with numerous health benefits [1]. However, globally, children are failing to meet the recommendations for physical activity [2], including in Wales [3].

Study into the relationship between motor development and lifelong physical activity is not new, with early work by Seedfeldt (1980) proposing the notion of a proficiency barrier in middle childhood if young children fail to achieve adequate levels of skill in their fundamental motor skills [4]. Clark and Metcalfe’s 2002 model highlights the need for a broad range of movement skills that they suggest form the base camp needed to climb a variety of mountains of motor development [5]. Further to this, the complex relationship between motor competence, perceived competence, fitness, physical activity and health was proposed by Stodden and colleagues in 2008, suggesting that early childhood is a critical window of opportunity to develop motor competence and support engagement in physical activity and entering a positive health trajectory [6]. Numerous studies have since examined this relationship and demonstrated the importance of motor competence in driving physical activity engagement [7,8]. Research has also identified associations between motor competence and a range of broader health outcomes, including healthy weight status [9,10] and cardiorespiratory fitness [11]. Studies have also linked motor competence to psychological constructs such as higher self-esteem [12] and perceived physical competence [13,14]. Jones et al. (2021) suggested associations between motor competence, cognitive development and school readiness [15], with Veldman et al. (2019) identifying gross motor skills as positively associated with cognitive development in toddlers [16]. Research with an existing proven motor development programme, Successful Kinaesthetic Instruction for Pre-schoolers (SKIP) highlighted that children in the SKIP intervention developed better fundamental motor skills and executive functions [17].

What is clear in developmental models and the existing research is the recognition of early childhood as a key window of opportunity for developing the foundational skills that will drive later physical activity [6,7,18]. As such, many motor skill interventions globally have focused on developing motor skills in early childhood and have been shown to have significant impact on motor competence. The evidence-based motor development programme SKIP was originally developed to address developmental delay in motor skills with children in areas of deprivation [19]. SKIP combines principles of motor development theory and physical education pedagogy, and multiple research studies across a variety of contexts have examined the impact of the programme. Studies have measured the impact of SKIP on motor skills [19,20], teachers and practitioners’ ability to deliver SKIP in Wales as SKIP Cymru [21] and in Indonesia as INDO-SKIP [22].

The need for successful motor interventions such as SKIP is clear with levels of motor competence shown to be at concerningly low levels [23,24,25,26]. With the clear links between motor competence and physical activity, multiple programmes have been put in place to address this through professional development in schools, pre-schools, community settings and the home [23,27,28,29].

However, parents are also key influencers of children’s physical development and studies have shown that with support, parents can improve their children’s fundamental motor skills (FMS) [30]. The important role that parents play in supporting children’s development has been acknowledged in both research and policy with the Welsh Government launching their ‘Education begins at home’ campaign in 2014 and further developing support materials for parent engagement highlighting that parent engagement is a powerful influence on school improvement. Drawing on the research of Desforges and Abouchaar (2003) [31] the government guidance suggests that the degree of family engagement can have “six times more influence over the child’s educational attainment than the quality of the school” [32] (p. 5). Further guidance for organisations to support working with parents was published in 2017, also highlighting the important role that parents play in their children’s development [33]. However, in all this guidance, there is scarcely any mention of physical development or how parents can support their children to develop the vital motor skills that underpin their physical development other than a brief mention of physical activity in the context of weight management [33] (p. 154).

Whilst there is recognition of the important role parents play in supporting their children’s development, including some research involving motor development [30], Agard and colleagues highlight concerning gaps between theory and practice in relation to parents’ understanding of early years physical activity [34] and there are limited interventions that target the home environment [35]. The Brian et al. (2023) SKIPing with PALS motor intervention with parents demonstrated increased competence for the children when parents engaged in more sessions [36]. Considering the global concerns surrounding childhood inactivity [2], poor motor skills [37] and overweight and obesity [38], there is a growing need for action to address these issues beyond school and pre-school and empower parents to support their children’s motor development at home and in their communities. Flynn et al. (2023), in a recent review of parent engagement studies, found that parental involvement in interventions significantly improved the FMS of children [39]. However, parents reported many barriers to supporting their children’s physical development, including environmental, time and energy constraints [40]. Brian et al. (2023) highlighted the potential of mobile applications (APPs) to support parent engagement with their child to promote physical development [36]. It is also highlighted that using mobile APP technology offered the potential to empower parents and support them by incorporating in the APP to promote their children’s physical development in their own home and local community [39] and saw significant improvements in both locomotor and object control skills [39,41,42]. The affordances and experiences of the home are significantly related to children’s motor competence [43], therefore supporting parents to enhance the affordances and activities in their home as an opportune way to support motor skills in children. The use of technology to provide developmentally appropriate activities for parents and children in an easily accessible and instructional format could support parents globally. Such an approach could have widespread impact on enhancing children’s physical development before entering the school system and serve as a foundation for work conducted in schools and pre-schools.

As a result, the MiniMovers (MM) APP (MiniMovers.org, accessed on 9 December 2023) was developed to address global concerns about children’s developmental delays in motor skills and provide support for parents. The MM APP draws on over 30 years of motor development evidence from the SKIP motor skill programme to design activities that are at an appropriate level for the children engaging in the activities with their parents. Combining motor development expertise with creativity expertise, the MM APP provides resources to parents to support their children’s motor competence with developmentally appropriate activities and equipment. The MM APP has three levels representing Mini, Mighty and Mega movers that represent different levels of a child’s motor competence. Built into the APP motor development experts designed an algorithm that asks parents about their child’s age and questions about some of their child’s physical skills such as “can your child catch a small ball with their hands?” Resulting from these data, the MM APP then places the child into one of the three levels (Mini, Mighty, Mega). At each level, there are four creative worlds (farm, sea life, jungle, space) with eight activities in each world that are developmentally appropriate for that level of motor competence. This ensures the children can engage with activities at the appropriate level for their stage of physical development and as such, it aims to ensure the children are engaged, experience success and make progress in their motor competence. For each activity there is a video of a parent and child performing the activity with music for motivation and animated characters relative to the world. In addition, the APP provides information to parents on how to set up the activity, the equipment needed, cues to support their child and how the activity is supporting child development.

This study was the first exploratory step in a line of future MM research to examine whether parents could utilise the MM activities at home and how parents and children responded to these activities. Additionally, to inform future research it was important to explore a variety of lab- and field-based assessment measures to determine what measures best captured changes in child motor skill outcomes as a result of the MM programme. Thus, the overall purpose of this study was to examine the feasibility and effectiveness of an 8-week MM programme on children’s motor competence. This initial pilot study addressed the following research questions: (1) What is the feasibility and experiences of families using the MM programme? (2) What is the impact of the 8-week MM programme on children’s motor skill performance? (3) What assessment methods best discriminate changes in motor performance as a result of the MM programme?

## 2. Materials and Methods

A mixed-methods design was utilised to evaluate the effectiveness of the 8-week MM programme on children’s motor competence and family’s experiences of using the MM activities. As this was both an initial feasibility study and small pilot study it was believed that both quantitative and qualitative data would help inform further development of the APP and future research. As this was an exploratory study, a wide variety of lab and field measures of motor skill competence were utilised to inform future research. Quantitative data consisted of 9 pre–post-test measures of motor competence to evaluate the effectiveness of the MM programme on motor competence. Qualitative data consisted of parent questionnaires every two weeks and semi-structured interviews at the end of the programme.

### 2.1. Context and Participants

A convenience sample of six families were recruited from Southwest Wales using professional networks from the Wales Academy for Health and Physical Literacy (https://www.uwtsd.ac.uk/wahpl/, accessed on 9 December 2023). Due to ongoing COVID-19 restrictions at the time of this study, families had to be able to attend the lab for testing, so the sample was determined by this. The six families with eight children completed both the pre- and post-tests. Table 1 provides demographic and anthropometric information on the children (*N* = 8) tested. No children were overweight (>91st centile) or with low BMI (<9th centile) [44,45].

### 2.2. Instrumentation and Qualitative Data

#### 2.2.1. Motor Competence Tests

Children performed 9 motor tasks prior to and following the 8-week MM programme. For many of the tasks, multiple variables were calculated in order to provide a broad array of data to inform future research. The reason so many variables and measures were utilised is that we were trying to identify what measures best captured potential changes in motor competence as a result of the MM programme. The testing took place at the Assistive Technologies Innovation Centre (ATiC) lab which was equipped with an 8-camera motion system (Simi Motion, Germany; sampling at 200 Hz) for motion capture and analysis along with two GoPros (sampling at 60 Hz) and 4 observation cameras (Noldus, the Netherlands; sampling at 30 Hz). Kinematic data were filtered by a second order lowpass Butterworth filter with a cut-off frequency at 8 Hz. The lab also included a force plate (HUR-FP8, HUR, Finland) with software (version: 2.65.4.0) for vertical jump (sampling at 1200 Hz) and for stand and one leg balance (sampling at 100 Hz). MATLAB R2020a (Mathworks, USA) was also used for calculating balancing parameters. Mighty and Mega children wore an eye tracker (Tobii Pro Glasses 2, Tobii, Sweden; sampling at 50 Hz) in catching (the glasses did not fit smaller children). The videos were able to show the reason children failed to catch the ball, e.g., did not secure the ball in hands or intercepted it too late. The eye tracker and the motion capture system were synchronised by common events in the video of both systems. Table 2 provides an overview of the 9 motor competence tests and variables that were evaluated pre- and post-test.

**Table 2:** Lab-based measures of motor skills.

Task: Instruction [Preparation]	Parameters
**Run**: run as fast as possible for 10 m [Starting and finishing lines marked on floor]	Time: time from the start of running to reaching the finishing line Mean velocity: 10 m divided by running time (seconds) Maximum velocity: fastest velocity of the marker located at pelvis level (mid spina iliac posterior superior) in the running direction Step length: average of the last 4 steps towards 6 m
**Underarm and overarm throw**: throw the ball as far as possible [Tape measure, marking stickers, rubber Koosh balls]	Throwing distance: distance between the front toe and the object’s landing position Release height: maximum height of the object centre over the floor Scaled release height: release height divided by child’s body height Release velocity: resultant velocity of the wrist at the ball-releasing moment
**Horizontal jump**: jump as far as possible (from standing position) [Tape measure, marking stickers]	Jump distance: distance between the starting line and the heel’s landing position Scaled jump distance: jump distance divided by body height Take-off velocity: resultant velocity of the back pelvis marker at the take-off moment. When two feet did not take off simultaneously, the midpoint of the two moments was defined as the take-off moment Take-off angle = ArcTangent(Vz/Vy) ·180/π, where Vz and Vy were the velocity along Z and Y axis, respectively (see Figure 1 for global axes)
**Vertical jump**: jump as high as possible (from standing position on the force plate) [Force plate]	Jump height (via impulse-momentum method), take-off velocity, maximum power, maximum impulse, maximum force/weight: measured by force plate software
Kick: kick (a deflated ball) hard towards a “gate” [Inflated ball]	Maximum velocity of the outer ankle bone calculated in 3D space
**Stand balance**: stand still (on the force plate) for 30 s [Force plate]	Duration: valid captured standing time in the 30-s trial. If >30 s, count as 30 s C95 Area: 95% confidence area of centre of pressure (COP) ellipse Normalised C95 Area: C95 area divided by duration COP velocity: total COP trace length divided by duration
**One leg balance**: stand on one leg (on the force plate) for 30 s (could start over if the leg touched the force plate) [Force plate]	Same parameters as for the stand balance
**Catch**: catch the ball (5 tosses thrown to chest from 7 feet). Based on performance, harder (thrown to side) or easier (rolled on the floor). Catch with and without the eye tracker. [Eye tracker, 10 cm rubber ball]	Success rate: the % of successful catches (from 5). The additional harder catching was not reported in the success rate Eye latency: time of the gaze velocity exceeding 30 deg/s after the ball started moving upwards and towards the child (trial start) Hand latency: time of the wrist velocity exceeding 10 cm/s after trial start Lag = hand latency − eye latency Trial duration: from the trial start to when the hand touched the ball. If the hand totally missed the ball, it would be from the trial start to when the ball dropped to the hands’ overground level.

All tasks were demonstrated by a research team member before a child completed the task. In each task (except catching), we aimed to collect two successful trials with the better performance reported in the results. When they had accomplished a task, children received a star sticker as motivation and at the end of the testing received a medal.

#### 2.2.2. Test of Gross Motor Development 3 (TGMD-3)

The skill criteria from six of the skills in the TGMD-3 [46] were utilised to evaluate the pre- and post-test process motor performance of the: run, horizontal jump, underarm throw, overarm throw, kick, and catch. Children had a practice trial and then performed two coded trials of each skill, and criterion elements of form were coded off the video resulting in a total skill score (number in parentheses represents total possible score for the skill): run (8), horizontal jump (8), underarm throw (8), overarm throw (8), kick (8), and catch (6). Two expert coders evaluated the participants’ performance from the video and an inter-rater reliability was established at 98%. These skills were evaluated at the pre- and post-test.

#### 2.2.3. Stages of Run, Horizontal Jump, Overarm Throw, Kick and Catch

In addition to the TGMD-3, the investigators evaluated the developmental stages (number in parentheses represents the total number of stages) of the: run (4), horizontal jump (4), overarm throw (5), kick (4), and catch (5) as the best representation of where a child is at developmentally in the emergence of a skill. The total body approach to the run [47,48], horizontal jump [48,49], overarm throw [48,50], kick [48,51], and catch [48,50] were used. In the total body approach to developmental stages, descriptions of movement patterns are placed in order from crude and inefficient patterns of movement (stage 1) to more mechanically efficient and proficient forms of movement (stage 4 or 5). Each stage in the sequence describes the common patterns of movement performed by children as they learn the skill and is representative of developmental level.

### 2.3. Qualitative Data

#### 2.3.1. Two-Weekly Questionnaires

Throughout the programme parents were sent questionnaires every two weeks to gain initial feedback on the activities that they had completed in those two weeks. The questionnaire collected data on where and how often they had played the MM activities, what equipment they had used, what they thought of the activities, how the children responded, any changes that they made to the activity and challenges they encountered. The qualitative data from these questionnaires were collated and analysed in conjunction with data from the semi-structured interviews.

#### 2.3.2. Interviews

At the end of the 8-week programme semi-structured interviews were conducted with the mothers (*N* = 5) of the children who completed the programme both online using Microsoft Teams and face-to-face depending on the preference of the interviewee (one mother missed the interview due to work commitments). Drawing on literature in the field and the project’s aims, an interview guide was developed and used to structure the interviews with parents to ensure consistency. Questions encompassed parent understanding of their child’s physical activity and motor competence, engagement with the activities, experiences of social interaction and general thoughts of the experience. Interviews were recorded and transcribed for analysis.

### 2.4. Procedures

This study was conducted in accordance with the Declaration of Helsinki, and approved (EC934, 11 October 2021) by the Ethical Committee of University of Wales Trinity Saint David (UWTSD). Written consent forms were obtained from all parents participating. Once parental permission was acquired, child assent was obtained and continued throughout the programme and the two testing days using developmentally appropriate procedures.

#### 2.4.1. Pre-Test

**Figure 1:**
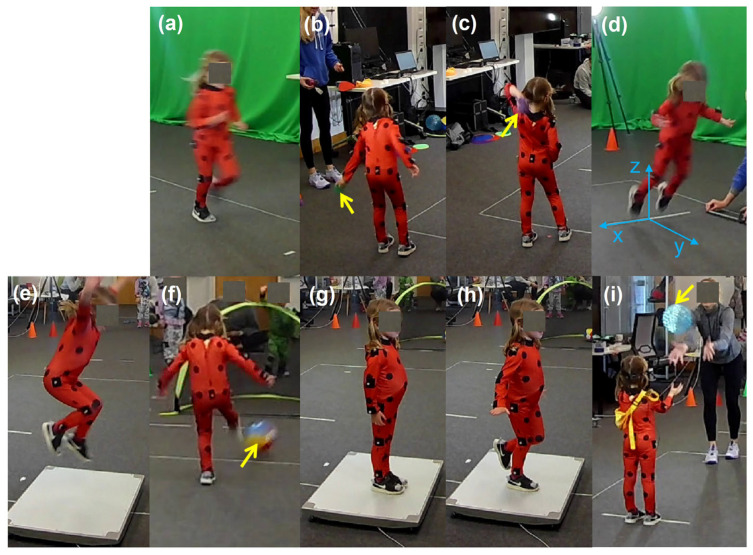
Task illustrations following the actual testing order. (**a**) run; (**b**) underarm throw; (**c**) overarm throw; (**d**) horizontal jump; (**e**) vertical jump; (**f**) kick; (**g**) stand balance; (**h**) one leg balance and (**i**) catch. Yellow arrows indicate the location of the ball.

**Figure 2:**
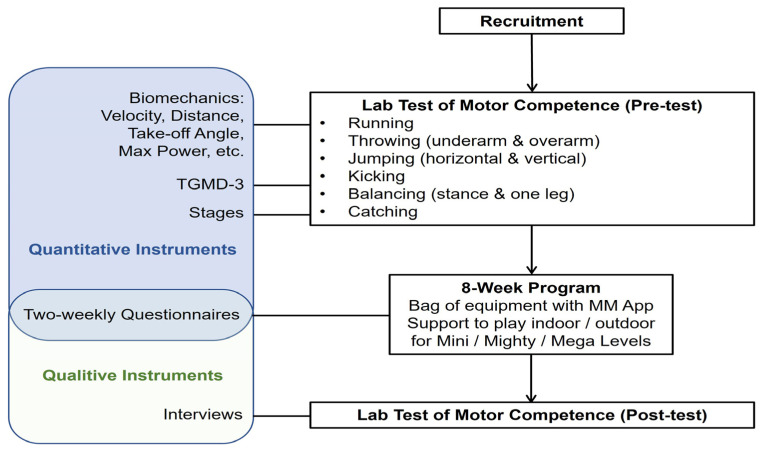
Diagram of the procedures, including pre- and post-tests and the quantitative and qualitative tools used in this study.

Figure 2 shows the procedures of the project. After the participants were recruited and ethics were completed, families were invited to the lab for pre-test measures. Children were changed into Lycra superhero dress-up suits to make the activity fun. Reflective markers were connected using Velcro at the: mid spina iliac posterior superior, great trochanters, lateral knees, outer ankle bones, foot second toe tips, heels, shoulders, lateral elbows and wrists (see Figure 1). The 9 motor skill tasks were evaluated via marker-based motion tracking, force plate and eye tracking (see details in Section 2.2.1). The movement trials for the TGMD-3 and developmental stages were videotaped and coded off the video.

#### 2.4.2. The MiniMovers Programme and Post-Test

After the pre-test, children were assigned to Mini (*N* = 3), Mighty (*N* = 2) or Mega (*N* = 3) levels and activities based on their stage of motor development. Parents were given the MM bag of equipment (balls, bats, poly-spots, bean bags, scarves, etc.) to take home to conduct the MM activities. Families participated in the 8-week MM pilot programme following the MM activities which included video and instruction demonstrations. The activities were a mixture of parent–child games that targeted the development of locomotor and object control skills across the 8 weeks. Two-weekly online questionnaires were completed by parents and the research team contacted them weekly to check in. At the completion of the 8-week programme, the children returned to the lab to complete the same battery of tests as the pre-test. After the post-test, parents completed the semi-structured interviews (*N* = 5) reflecting on their experiences of the programme.

### 2.5. Data Analysis

#### 2.5.1. Quantitative Data Analysis

Statistical tests were carried out using SPSS 28 (IBM, Armonk, NY, USA) and RStudio 1.3 (PBC, New York, NY, USA). Normality (via the Kolmogorov–Smirnov test) was checked first for all performance parameters. The pre–post-test lab measures (see Table 2) were evaluated using paired-samples *t*-tests for normally distributed parameters. In addition, as the sample size was so small and the variability of the data was high, we used a method with a covariate (coded in RStudio) to remove the partial added error variance by a paired sample *t*-test, which enhanced the sensitivity of the test and therefore increased test power likelihood [52]. For other non-normally distributed data (e.g., the catching success rate and one leg balance C95 area), the non-parametric Wilcoxon signed-rank test was used. For field measures, we used a pre–post-test ANOVA to examine differences in the six skills of the TGMD-3. For the five skill stage data (which is ordinal), we used a Wilcoxon Signed Rank test. The significance level was set at *p* < 0.05.

#### 2.5.2. Qualitative Data Analysis

Interviews were transcribed verbatim. Qualitative data from the questionnaires were combined with the interview data and analysed using thematic analysis which is widely used in qualitative sport and exercise research [53,54,55]. Drawing on the work of Braun and Clarke (2006) [53], the analysis comprised of six phases outlined in Table 3.

## 3. Results

### 3.1. Quantitative Data

#### 3.1.1. Findings of the Nine Motor Tasks

Height and Weight—After 8 weeks, the children were taller by 0.7 ± 0.8 cm and heavier by 0.4 ± 0.6 kg. Table 4 shows the pre- and post-test performance of the nine motor tasks including the findings for the paired sample *t*-tests (*p*) and paired sample *t*-test with the covariate (removing the partial added error variance) applied (*p*’) [52]. The findings in Table 4 report significant pre- to post-test differences for: 1) run time, 2) underarm throwing distance, 3) horizontal jump distance, and 4) horizontal jump distance scaled by height (all at *p*’ < 0.05). In the 10-m running task, the children significantly reduced their time by 0.5 s. Run mean velocity and step length increased (0.2 m/s and 2.7 cm) and neared significance. However, maximum run velocity only decreased by 0.2 m/s and was not significant. Underarm throwing distance significantly increased 1.5 m from pre- to post-test. However, none of the other underarm and overarm throwing variables improved significantly despite mostly showing improvements in the data. Jump distance significantly improved 10 cm and when scaled by height children went from jumping 66% of their height to 77%. Vertical jump showed minimal/no improvements and no variables were significant. None of the kick or balance variables were significant and there were little/no improvements in these measures. For catching, the hand was more prolonged in its latency in the post-test, probably affected by the significantly longer trial duration. The eye latencies for children at the Mega level (averaged pre and post: 227 ms) were shorter than those at the Mighty level (300 ms). In order to accommodate for the small sample size, it was beneficial to adjust the *p* level by the covariate to improve the sensitivity of the test and also increase the power of the statistical tests. For example, the power increased from 0.49 to 0.99 for running time, and from 0.36 to 0.80 for horizontal jump. Except for underarm throwing distance and eye and hand latencies, the power was similar.

**Figure 3:**
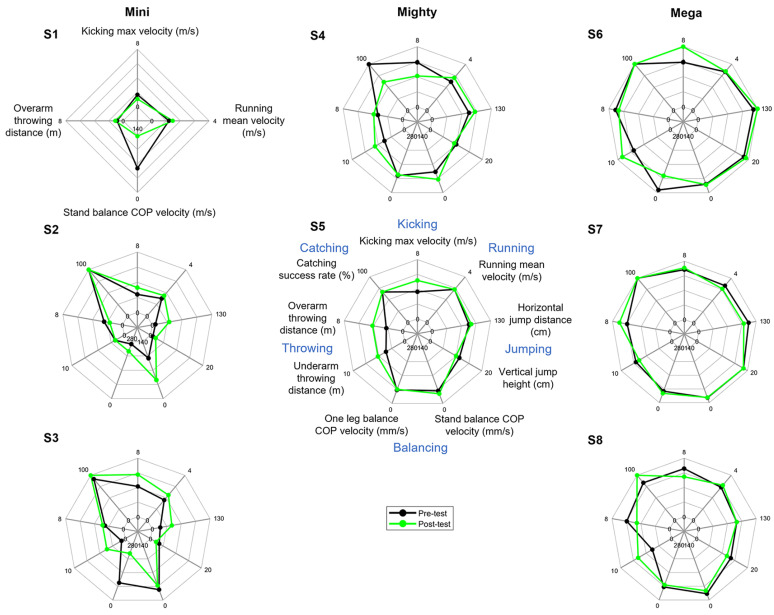
Individual performance of pre-test and post-test. Each axis represents a parameter taken from each task, with the expanding direction as a better-performance direction. The three columns of spider plots represent data of Mini, Mighty and Mega levels, respectively, with an order of increasing age from top to bottom (Child ID: S1–S8, see Table 1). The youngest girl (S1) at the Mini level was not able to perform all the tasks.

As this was an exploratory study and had a small sample size, we decided to present each child’s performance in an individual spider plot (Figure 3). A larger spider plot area designated a child with better skills. In terms of the area size of the pre- and post-test performances (presented in black and green), S2 at the Mini level and S5 at the Mighty level had a larger area, i.e., an overall improvement after the 8-week program. For S7 and S8 of the Mega level, the area size changed very little. This was similar to S3 and S4, but these two participants had more tasks that showed an improvement. S6 at the Mega level also increased the vertical jump, long jump and kicking, all tasks that require lower leg strength. S3 also improved in almost all tasks (kicking, throwing, running and jumping), but not balancing. Overall, five children improved across different ages, with two children improving in almost all tasks, and three children had more tasks improved than did not.

Only S4 had a decreasing success rate when catching a ball. In some catching cases, the child intercepted and touched the ball but was not able to secure it and the ball dropped. We saw evidence of this in S4 at the post-test and if she had been able to secure the ball in her hands it would have changed the post-test success rate from 62% up to 85%. Four children maintained their successful catching rate, either 100% (S2, S6, S7) or 67% (S5). Two children caught the ball better, with a success rate of 8% and 17% higher than the pre-test (S3 and S8, respectively). Apart from catching, S4 did not improve in kicking either in the post-tests. However, she could jump farther than the pre-test. She was also better at running, throwing and balancing.

**Figure 4:**
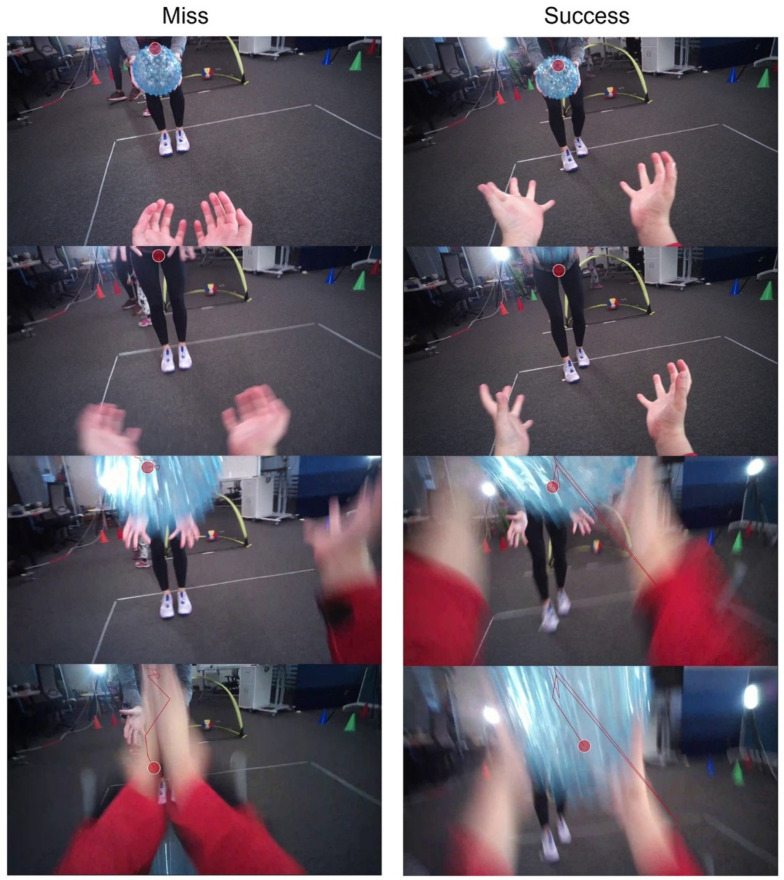
A failed and successful catching trial of eye tracking during ball catching.

Other than successful interception but without a firm catch, the failed catches could be sometimes caused by inappropriate timing of the interception. The eye-tracking data in Figure 4 revealed some interesting findings. This example shows the hands in the view of a child (Mighty level) in the pre-test, and the frames from top to bottom shown are the moments right before the ball was thrown, after the ball started moving (gaze point was left behind), when the gaze was on the ball again, and the hands trying to intercept the ball at the end. The late hand interception with the hand shown in the eye-tracking view from the child’s perspective, demonstrated the gaze behaviour was similar between a trial of miss and success. In the left panel where the child did not successfully catch the ball, the hands did not move in time to the ball, so the ball passed through the space between hands (i.e., the hands intercepted too late).

#### 3.1.2. Findings of the TGMD-3 and Stages

A pre–post-test ANOVA was conducted to examine changes in the TGMD-3 skill scores across the 8-week MM programme. Table 5 provides the pre- and post-test TGMD-3 data. All skills except for catching demonstrated a significant gain in scores as a result of the MM programme. There was high variability in the scores among participants with SDs being very high.

A Wilcoxon Signed Rank test was conducted to examine pre- to post-test changes in the developmental stages of the overhand throw, catch, kick, run, and horizontal jump. Table 6 shows the pre and post-test means and *SD*s of the five skills along with the *p* level. Catch, kick and run showed significant improvements in skill scores with overhand throw and horizontal jump close to significance. As with the TGMD-3 data, there was high variability in the scores among participants.

### 3.2. Qualitative Data

In the four questionnaires, parents reported a simple measure of feasibility indicating how many times per week they engaged in the MM activities with their children. In weeks 1–2 the *M* = 4.63, weeks 3–4 *M* = 4.75, weeks 5–6 *M* = 4.75 and weeks 7–8 *M* = 5.50 (range was 3–7 days per week for all weeks except the last two weeks where it was 3–8) with an overall average of 4.91 activities per week per child. Analysis of data from the semi-structured interviews and the qualitative data from the questionnaires were analysed drawing on six phases of thematic analysis as outlined by Braun and Clarke (2006) [53].

Overall, three qualitative themes emerged around: (1) enjoyment, (2) independence, and (3) parents reporting their knowledge improved and subsequently their ability to identify their child’s progress and success in performing the MM tasks. Table 7 presents the three themes and gives examples from the data and the process of analysis that led to the themes. The codes are examples of initial coding steps that were further refined and combined to sub themes before the final stage of analysis into the three main themes. The themes will be discussed in depth in the following section.

## 4. Discussion

There is paucity of literature around parent engagement to promote their child’s motor competence [39], yet we know that parents are the primary role models and gatekeepers of young children’s physical development. This initial feasibility and pilot study demonstrates the promise of the MM APP programme to empower parents to support their children’s physical development. The overall purpose of this study was to examine the feasibility and effectiveness of an 8-week MM programme on children’s motor competence. There were three research questions.

### 4.1. Feasibility and Experiences of Families Using the MM Programme

Parents were consistently playing the activities with an average of 4.91 times per week over the 8-week period reported in the questionnaires. Whilst we have frequency of engagement, we do not have the dose as the parents did not report how long they played the activities. The data does show number of times of engagement across the 8 weeks ranging from 4.63 to 5.5 times per week. This is encouraging as it demonstrates that the engagement was consistent throughout. Of particular interest was that the highest mean engagement was in the last two weeks when we may have expected the families to be getting programme fatigue. This may be explained by the qualitative findings which highlighted enjoyment as a key theme. Parents highlighted in both the questionnaire feedback and the interviews how both they and their children had enjoyed the MM APP activities. Comments noted enjoyment of specific activities with one mother stating “The obstacle course was fab. I enjoyed setting it to challenge my kids” (M4) and another echoing this “The obstacle course was great fun and easy to set up in the house” (M5). The parents noted the ease of setting up and playing the activities “Easy to set up, I like the fact I can say, shall we do MiniMovers and we have the stuff there and ready to go and we are playing within minutes” (M6), “I’ll go, right we’re going to do like mini movers and it’s just easy to set up because I’ve got the videos to show them” (M4). The ability to set and play the activities easily removed the barriers highlighted by Bentley et al. (2012) [40]. Parents also noted their children’s enjoyment of playing games with them, “she loved it! Especially when she was moving the spots further away from each other to make it harder for her dad to hop between” (M3) and they spoke of their own enjoyment of playing with their children “really fun and engaging for both me and the kids” (M4). Parents noted that it was a good way for more social interaction with their children, “she wants to do it together” (M3) and of being less sedentary “we kind of we get in the habit of putting the telly on in the evenings, but it’s a good way of not doing that” (M3). It is paramount that families enjoy the APP activities if they are going to continue to engage and, as Flynn et al. [39] suggest, improve their children’s motor skills.

Another key theme highlighted in the data was independence. Parents noted how quickly children were able to learn the activities and then set them up independently “He would also play it without me initiating” (M4) “they’ll just help themselves, and they’ll set things up” (M4). The videos meant children were able to learn the activities and have more control in their playing “having the videos there makes it easier for her and means she’s got a bit more control over things herself” (M5), “She put them out in different places” (M1) and “she did decide to add extra obstacles in such as cushions and the sofa” (M5). Parents also reported children adapting the games and creating their own versions “he made the river with a scarf and did little stepping stones in the river that he did all that by himself” (M2). This ability for the children to learn activities and then go onto to practise them gives more opportunities for physical activity to drive motor competence [6] which in turn will drive later physical activity [7,8]. This is of particular importance for children of this age as this is a critical window of opportunity for laying foundations for lifelong physical activity, health and psychological outcomes [6,7,8,9,10,12].

The final theme to emerge was parent knowledge. It was clear that parents did not know that they needed to support their children to develop motor competence prior to engaging in this study and they valued the increased understanding of how they could support their children “For like a minute we’ll just practise catching, which is something I wouldn’t necessarily have done before because I didn’t realise how important it was” (M4). The parents were spending more time observing their children and were able to note improvements in their ability “her hopping and control/balance really improved through the game” (M3) and were getting learning to alter the equipment “It was maybe a bit hard but on advice from the research team I tried a softer ball” (M4), and they were altering the activities as children progressed such as “lots of minor adaptations to the obstacle course” (M3) and “we’ve used scrunched up paper as extra balls and lightly scrunched paper and stuff” (M5). As they progress through the 8-week programme, parents were able to observe their children and note that changing the equipment impacted their children’s success—“and then we try different balls and it’s, I think really worked” (M4 week 8 feedback). These data suggest that parents’ knowledge of their children’s progress has enabled them to enhance the affordances and experiences in the home, which Flôres et al. (2019) identify as being significantly related to motor competence [43]. Parents were pleased to be able to identify when they felt their children had made progress with one (M1) noting the impact of motor improvements on her daughters confidence and “these games have really benefited her cause her balance is so much better then her confidence like the other day, I caught her and she’s been so risk averse I caught her trying to climb something and she was so chuffed because she could climb it” and M2 noting “I think that he’s come on really well. Yeah, yeah, he’s definitely improved”. These observations align to existing research which highlights the relationship between motor competence perceived competence [13,14] and self-esteem [12]. With concerns about the lack of parents’ knowledge in relation to early years physical activity [34] and recommendations for using APP technology to support parents [36], the MM APP offers some initial positive findings. Future research needs to explore in more depth the user experience of the APP drawing on theories of behaviour change, which is beyond the scope of this study.

### 4.2. Influence of the 8-Week MM Programme on Children’s Motor Skill Performance and Best Measures to Discriminate Child Outcomes

Fundamental motor skills (FMS) form the base camp to the mountain of motor development [5] and are critical to future engagement in physical activity [6,8]. FMS consist of locomotor skills like running and jumping and object control skills like throwing and kicking [48]. Early childhood is the window of opportunity in which to develop these skills [5,48]. The MM activities incorporate a wide variety of locomotor and object control activities in developmentally appropriate and engaging settings using music and creativity (different worlds) and parents as facilitators. The activities that are incorporated in the MM APP are founded on over 30 years of SKIP research across a wide variety of contexts and countries [17,20,21,36]. Thus, a strength of the MM APP is that the activities themselves have a strong evidence base. What is not clear from the research to date is whether parents can bring about meaningful changes to their children’s motor skill performance in their own homes. This study attempted to provide a very initial response to that question as both a feasibility and pilot study of the influence of the MM programme on children’s motor performance.

We used a large number of lab- (see Table 2) and field-based (TGMD-3 and stages) measures of motor performance to examine the influence of the MM programme on the motor performance of the children. This study was exploratory in nature and the reason we undertook so many measures was to inform future research in this area. This work was partially funded by an Accelerate Wales programme that gave us access to the ATiC lab with a wide range of technologies such as an 8-camera motion capture system, GoPros and a force plate and thus the ability to undertake more sophisticated measures of movement. However, it was interesting that few of these measures resulted in significant pre-to post-test improvements in motor skills. Run time, underhand throw distance, jump distance, jump distance scaled by height, catch lag and catch duration were the only significant findings. We did not expect the difference in catch duration as it suggested the throwing conditions were different and could affect the hand and eye latencies (a shorter trial might require a child to respond more quickly). Although we calculated these measures using the kinematic data, run, throw and jump could have easily been conducted using a stop watch and measuring tape. In all measures the small sample size and large standard deviations made it more challenging to find significance.

Overall, two locomotor skills, run and horizontal jump demonstrated significant improvements in multiple measures of the skills. For run, the TGMD-3, stage data and run time demonstrated that the MM programme resulted in significant improvements to running process and product measures. Additionally, run velocity and step length neared significance. For horizontal jump, the TGMD-3 and jump distance plus jump distance scaled by height were significant. Children improved their jumping from 66% of their height to 77% which is quite substantial. Both skills require leg strength and multi-limb coordination and it was heartening to see that the MM activities were helping children develop in this area. All children struggled with the vertical jump and this was not considered to be a good measure for children this young. Overall, we concluded that run and horizontal jump (scaled by height) are two good measures for future research. Both are quite simple to measure. However, it should be noted that the 0.5 sec decrease in run time may not be able to be detected so well using a stop watch versus coding off the video.

For object control skills overhand throw, underhand throw, kick and catch were the skills that emerged as having significance. The only measure of overarm throw that was significant was the TGMD-3 going from 0.14 to 1.43 critical elements out of a possible 8 points (four critical elements by two trials). Interestingly, the stage data were not significant, with children going from 1.71 to 2.57. We suspect with a larger sample size we would find significance in this variable. For underarm throw both the TGMD-3 and throw distance significantly improved with children going from 2.43 critical elements to 4.71 out of a possible 8 points. It was good to see these throwing data as there were lots of activities in the MM programme that worked on throwing. For kicking both the TGMD-3 and stage data significantly improved. Children went from less than one critical element (0.71) to 4.71 points and from a stage 1–2 (M = 1.57) to a stage 2–3 (M = 2.86) meaning that children went from a stationary kick to a moving approach to kicking the ball. This is quite remarkable given the fairly small dose of the MM programme. Both parents and children noticed these improvements in kicking skills with one parent (M4) commenting that her child had noticed she got better and she was going to put her child in football as a result of the success and fun in the kicking activities. For catch, children significantly improved their stages going from 3.14 to 3.71. Surprisingly the TGMD-3 data did not reveal a significant improvement going from 3.86 to 5.14, probably in part due to the large standard deviations.

For catching, the trial duration was significantly longer in the post-test, with the hand more prolonged in its latency. This may have been due to the instructor who was different between pre-test and post-test so the distance, speed and trajectory were mostly likely different. Future research needs to think about the delivery of the ball in catching and try and standardise the toss to the participant. The eye-tracking data revealed some interesting findings about why the children were failing to catch suggesting that sometimes their hands were late to intercept the ball flight and on other occasions they made contact with the ball but did not secure the ball in their hands dropping it to the floor. We suggest that in addition to catching success rate, future research also code if a child make contacts with the ball but drops it as this may be an early marker of child success in catching.

For those only interested in catching, the eye tracking is a very useful tool to monitor eye movement behaviour. In our tests, children over 4 years were fine to wear the Tobii Pro glasses for catching. It has been reported that the latency of eye movements such as saccade and vergence decreases with age (4.5–12 years) in children [56]. Our results also showed that the eye latency decreased in children of the Mega level compared to Mighty level. The Mega children were also able to track and pursue the ball when it was higher in the air, even with the head movement. However, the post-test showed slightly longer eye latency for 4 out of 5 children (except S8), as well as longer hand latency for everyone. This may be largely due to the throwing conditions not being well controlled such as differences in the instructors who threw the ball during pre- and post-tests. If a more comprehensive assessment of many FMSs is part of the research plan, we are not sure that the time and energy necessary to secure the data is worth collecting given the findings. Thus, more field-based measures appear to be most discriminatory.

Overall, the MM programme resulted in significant improvements to children’s FMS. We recommend the use of the following measures in future research. For locomotor skills run and horizontal jump are good skills to evaluate from both a process (TGMD-3 and stages) and product (time, distance) standpoint. Future research should consider what other locomotor skills like hopping might be of value to measure. For object control skills we recommend measuring overhand and underhand throw, kick and catch using both process (TGMD-3 and stages) and product (throw distance, catch success, catch touches of ball) measures of the skills. Additionally, the spider plots were good visual representations of change, especially when children have such large variability in their scores, and we would recommend using this approach in future research. The Mini (younger) children were quite challenging to test and we recommend that future research focus on the Mighty and Mega movers.

### 4.3. Limitations

The small sample size and large standard deviations are a major limitation of this paper. With larger sample size, a control group could be added to indicate the contribution of the MM programme and the results could also be compared at various MM levels (with smaller variation presumably). The standard deviations were particularly large in children in the Mini group and this group of three children were quite challenging to test needing many breaks and refocusing across the testing period. However, the significant findings across multiple skills and measures of motor competence provide a robust measure of the impact of the MM programme. We recommend limiting testing to the Mighty and Mega children. Due to the small and initial nature of this study we had a very limited measure of feasibility only knowing how many days each week parents performed activities through the questionnaires. Future research needs to add additional measures of fidelity and feasibility by including what activities the children played, for how long, and how they modified them (if they did), in order to determine a better measure of dose. It would also be valuable to know how the MM programme influenced behaviour change in children and parents and in what behaviours. The next step in this line of work is to undertake a larger scale research to evaluate the MM APP (available as “Mini Movers” in APP stores) as an ecologically valid and accessible approach to promoting motor competence in young children in a family environment.

## 5. Conclusions

This pilot study indicates how the MM programme enabled parents to support their children’s physical development and effectively improved their FMS. Moreover, the use of multiple measures enabled us to recommend approaches in the future to best discriminate changes in motor skills. Above all, it provides insights into the feasibility of the programme, which helps the future development and evaluation of the activities and the APP to support home activities among families with young children.

## Figures and Tables

**Table 1:** Demographic and anthropometric information of children.

Child ID	MM Level	Gender	Age (Months)	Height (cm)	Weight (kg)	BMI (kg/m^2^)	BMI Centile ^1^
S1	Mini	Girl	21	84	12	17.0	75–91
S2	Mini	Boy	29	85	11.9	16.5	75
S3	Mini	Boy	29	93	14.9	17.2	75–91
S4	Mighty	Girl	49	104	17.8	16.5	75
S5	Mighty	Girl	50	103	15.9	15.0	25
S6	Mega	Girl	73	116.5	21.9	16.1	50–75
S7	Mega	Girl	77	119	21.6	15.3	50
S8	Mega	Girl	79	109	17.4	14.6	25
Mean ± Standard Deviation		Pre	50.9 ± 23.4	101.7 ± 13.3	16.7 ± 3.8	16.0 ± 1.0	
		Post	52.9 ± 23.4	102.4 ± 13.0	17.0 ± 3.6	16.2 ± 1.2	

^1^ BMI Centile uses references of UK-WHO 0–4 years growth chart (for S1) [44] and BMI 2–20 years chart (for S2–S8) [45].

**Table 3:** Phases of thematic analysis drawn from Braun and Clarke (2006) [53].

Phases of the Analysis	Purpose	Process
Familiarisation	Immersion in data to gain an insight into the scope of the content. Gain a sense of meaning and patterns.	Transcription Deep reading Note making
Initial coding	Identifying initial codes in order to organise data	Labelling and grouping data
Initial themes	Sorting groups into initial themes relating to meaning. Examining relationships between themes	Sorting and mapping themes. Defining themes
Review of themes	Identifying patterns in the data and reviewing all data	Collapsing themes and checking data justifies theme
Defining themes	Identifying the meaning of theme and aligning to research questions	Re-checking data and themes—developing an audit trail
Presenting themes	Present concise themes	Write account of themes supported from data

**Table 4:** Pre-and post-test measures of the nine motor tasks.

Task	Parameter	N	Pre	Post	*p*	*p*’
Run	Time (s)	8	4.7 ± 2.0	4.2 ± 1.4	0.063	0.014 *
	Mean velocity (m/s)		2.4 ± 0.8	2.6 ± 0.7	0.128	0.069
	Max velocity (m/s)		3.2 ± 1.1	3.4 ± 1.2	0.495	0.499
	Step length (cm)		67.2 ± 25.1	69.9 ± 23.8	0.063	0.062
Underarm throw	Throwing distance (m)	7	4.0 ± 2.4	5.5 ± 2.4	0.026 *	0.036 *
	Release height (cm)		59.7 ± 7.8	66.0 ± 9.7	0.113	0.133
	Scaled release height (%)		57.8 ± 8.4	63.4 ± 10.7	0.133	0.166
	Release velocity (m/s)		5.1 ± 2.2	5.1 ± 2.5	0.994	0.994
Overarm throw	Throwing distance (m)	8	3.8 ± 2.2	4.0 ± 2.1	0.624	0.633
	Release height (cm)		97.1 ± 17.8	95.7 ± 17.9	0.615	0.636
	Scaled release height (%)		95.0 ± 7.6	92.8 ± 7.6	0.400	0.388
	Release velocity (m/s)		2.7 ± 1.3	3.1 ± 1.4	0.602	0.515
Horizontal jump	Jump distance (cm)	7	73 ± 44	83 ± 33	0.106	0.020 *
	Scaled jump distance (%)		66 ± 37	77 ± 24	0.113	0.011 *
	Take-off velocity (m/s)		1.8 ± 1.1	1.9 ± 0.7	0.869	0.700
	Take-off angle (deg)		22.9 ± 9.4	19.9 ± 4.4	0.466	0.154
Vertical jump	Jump height (cm)	6	12.0 ± 6.5	11.7 ± 6.4	0.545	0.586
	Take-off velocity (m/s)		1.5 ± 0.6	1.5 ± 0.5	0.973	0.967
	Max power (W)		490 ± 227	509 ± 241	0.284	0.325
	Max impulse (kg·m/s)		29.9 ± 12.5	31.0 ± 11.9	0.167	0.187
	Max force/weight (N/kg)		21.1 ± 1.1	21.9 ± 2.6	0.565	0.389
Kick	Max velocity (m/s)	8	4.6 ± 2.0	5.0 ± 2.2	0.494	0.521
Stand balance	Duration (s)	6	27.9 ± 2.5	29.8 ± 0.4		
	C95 area (mm^2^)		1266 ± 1276	1181 ± 490	0.839	0.605
	Normalised C95 area (mm^2^/s)		45.3 ± 42.8	39.7 ± 16.8	0.673	0.278
	COP velocity (mm/s)		16.6 ± 14.1	25.3 ± 8.7	0.759	0.663
One leg balance	Duration (s)	5	17.4 ± 9.9	16.0 ± 10.1	0.825	0.807
	C95 area (mm^2^)		1198 ± 551	1650 ± 720	0.500	0.308
	Normalised C95 area (mm^2^/s)		80.0 ± 47.2	160.6 ± 130.9	0.270	0.318
	COP velocity (mm/s)		58.2 ± 26.5	73.7 ± 15.9	0.333	0.155
Catch	Success rate (%)	7	91.7 ± 12.7	89.7 ± 17.6	1	
	Eye latency (ms)	5	248 ± 31	265 ± 81	0.646	0.696
	Hand latency (ms)		254 ± 46	401 ± 148	0.052	0.076
	Lag (ms)		6 ± 26	132 ± 102	0.044 *	0.086
	Trial duration (ms)		715 ± 69	876 ± 28	0.012 *	0.001 *

* *p* < 0.05. For the catching success rate and one leg balance C95 area, *p* was reported from the non-parametric Wilcoxon signed-rank test.

**Table 5:** Pre- and post-test measures of the TGMD-3 skills.

Skill (*N* = 7)	Pre-*M*	*SD*	Post-*M*	*SD*	*p*
Overarm throw	0.14	0.38	1.43	1.51	0.035 *
Underarm throw	2.43	2.15	4.71	2.43	0.007 *
Catch	3.86	2.85	5.14	1.57	0.093
Kick	0.71	0.95	4.71	2.22	0.001 *
Run	5.00	2.71	6.86	1.86	0.011 *
Horizontal jump	2.43	2.07	5.29	1.89	<0.001 *

* *p* < 0.05.

**Table 6:** Pre- and post-test measures of the stages for five skills.

Skill (*N* = 7)	Pre-*M*	*SD*	Post-*M*	*SD*	*p*
Overarm throw	1.71	0.95	2.57	1.13	0.063
Catch	3.14	1.57	3.71	1.60	0.046 *
Kick	1.57	0.54	2.86	0.90	0.024 *
Run	3.14	0.90	3.71	0.49	0.046 *
Horizontal jump	1.57	1.13	2.43	1.13	0.063

* *p* < 0.05.

**Table 7:** Summary of qualitative themes, data analysis and examples.

Theme	Sub Themes	Codes	Data Example
Enjoyment	Child enjoyment	Fun	“she’s been really enjoying it”
			“he does enjoy the music”
		Easy	“easy to do, and fun to do”
	Parent enjoyment	Together	“we’ve been able to look at them together”
		Activity	“it’s got me doing it Just trying to be more active with them as a whole”
Independence	Child independence	Self-organising	“she can set it up herself out-side”
	Child selecting and organising the activities		“she just likes experimenting a little bit”
		Child choice	“they’ll set things up”
			“she’s taking herself off and doing that”
Knowledge	Identifying progress	Observing	“I think her movements have become more fluid”
			“catching he’s still not great”
			“I think he his jumping is getting really good”
	Supporting children	Resources	“when I’ve put the spots on the floor, he can jump a lot further”
			“following the videos has really helped her”
			“I find getting him to watch the video really helps”

## Data Availability

The data presented in this study are available on request from the corresponding author. The data are not publicly available to protect the privacy of the participants.
